# Comparing genetic pathways variation of immunoinhibitory receptor LAIR-1 in murine vs human internal organs

**DOI:** 10.1186/1471-2105-15-S10-P9

**Published:** 2014-09-29

**Authors:** Shuqiu Sun, Yan Jiao, Wei Wei, Arnold E Postlethwaite, Weikuan Gu, Dianjun Sun

**Affiliations:** 1 National Center for Endemic Disease Control, Harbin Medical University, Harbin 150081, China; 2 Department of Orthopedic Surgery and BME, University of Tennessee Health Science Center, Memphis, TN 38163, USA; 3 Department of Medicine, University of Tennessee Health Science Center, Memphis, TN 38163, USA

## Background

Recent evidence suggests that leukocyte-associated immunoglobulin-like receptor-1 (LAIR-1) may play an important role in down-regulating immune activities upon collagen binding [1], and its defective expression or dysfunction is clinically associated with some autoimmune diseases [2-5], cancer [6-8] and viral infection [9-12]. The human genome encodes the counterpart to LAIR-1, soluble protein LAIR-2 [13], which also binds collagen and can interfere with LAIR-1/collagen interactions [14]. However, LAIR-2 has no homologue in mouse or rat [13]. To clarify the extrapolative credibility of a murine model to human disease, we compared LAIR-1 genetic pathways in internal organs of the two species.

## Materials and methods

### Data source

We used the GeneNetwork software developed by the University of Tennessee, and six eligible datasets of normal liver, lung, and brain tissue from the linked database [15].

### Statistical analysis

The top genes shared by mouse and human on the basis of Pearson correlation, were picked for plotting network graphs in GeneNetwork. An *r* absolute value >0.50 was considered to indicate connection line threshold.

## Results

### Significant variation in LAIR-1 genetic pathways was found in mouse vs human internal organs

The top 50 mouse genes by LAIR-1’s Pearson correlation were employed to search for the same genes in corresponding human tissue throughout relevant databases. There were 33 common genes found in liver, 32 in lung, and 31 in brain. The network node of LAIR-1 has a more robust connection for mouse than for human, and no common genes except for LAIR-1 were shared by liver, lung and brain in mice or humans. These observations can be confirmed in liver with the common genes of the top 100 human and top 100 mouse LAIR-1 relevant genes.

### Genetic interaction of human LAIR-1 with LAIR-2 *in vivo* rarely occurred

The interaction of LAIR-2 with LAIR-1 only occurred in the top 200 genes for lung tissue with a positive coefficient of 0.426, and in the top 300 genes for brain tissue with a negative coefficient of -0.242, but not for liver tissue, according to Pearson correlation distance and intensity.

## Conclusions

LAIR-1 genetic pathways have noteworthy species difference and tissue specificity, which may cause overestimation if using mouse experimental data to evaluate human conditions. Moreover, human LAIR-1 and LAIR-2 actually get the rare opportunity to interact *in vivo*, implying that species difference in regard to LAIR-1 genetic pathways could not be primarily attributed to the existence of human LAIR-2.
